# Characterization of Novel Pectinolytic Enzymes Derived from the Efficient Lignocellulose Degradation Microbiota

**DOI:** 10.3390/biom12101388

**Published:** 2022-09-29

**Authors:** Qin Miao, Xiaoling Zhang, Yitong Wang, Xiaoqi Li, Zheng Wang, Lingmin Tian, Lingbo Qu, Yongjun Wei

**Affiliations:** 1 Laboratory of Synthetic Biology, School of Pharmaceutical Sciences, Zhengzhou University, Zhengzhou 450001, China; 2 College of Life Science and Technology, Beijing University of Chemical Technology, Beijing 100029, China; 3 Department of Food Science and Engineering, Jinan University, Guangzhou 510632, China; 4 College of Chemistry, Zhengzhou University, Zhengzhou 450001, China; 5 Jiangsu Collaborative Innovation Center of Chinese Medicinal Resources Industrialization, Nanjing University of Chinese Medicine, Nanjing 210023, China

**Keywords:** pectinolytic enzymes, lignocellulose degradation microbiota, metagenomics, enzyme characterization, lignocellulose-degrading enzymes

## Abstract

Diverse pectinolytic enzymes are widely applied in the food, papermaking, and other industries, and they account for more than 25% of the global industrial enzyme demands. Efficient lignocellulose degradation microbiota are reservoirs of pectinolytic enzymes and other lignocellulose-degrading genes. Metagenomics has been widely used to discover new pectinolytic enzymes. Here, we used a metagenomic strategy to characterize pectinolytic genes from one efficient lignocellulose-degrading microbiota derived from pulp and paper wastewater treatment microbiota. A total of 23 predicted full-length GH28 and PL1 family pectinolytic genes were selectively cloned and expressed in *Escherichia coli*, and 5 of the expressed proteins had pectinolytic activities. Among them, the characterization of one pectinolytic enzyme, PW-pGH28-3, which has a 58.4% identity with an exo-polygalacturonase gene of *Aquipluma nitroreducens*, was further investigated. The optimal pH and optimal temperature of PW-pGH28-3 were 8.0 and 40 °C, respectively, and its pectinolytic activity at the optimal condition was 13.5 ± 1.1 U/mg protein. Bioinformatics analyses and structural modeling suggest that PW-pGH28-3 is a novel secretory exo-polygalacturonase, which is confirmed by its hydrolysates of polygalacturonic acid. The detection of PW-pGH28-3 and other pectinolytic genes showed that efficient lignocellulose degradation microbiota could provide potential efficient pectinolytic enzymes for industrial application. In the future, improving metagenomic screening efficiency would discover efficient lignocellulose-degrading enzymes and lead to the sustainable and green utilization of lignocellulose.

## 1. Introduction

Pectin is a natural biopolymer, and it is one of the main components of the plant cell wall [1]. Pectin is basically composed of D-galacturonic acid joined by α-1,4 glycosidic bonds; galactose, arabinose, rhamnose, xylose, and other simple sugars are available in pectin polymers [1,2]. Pectinolytic enzymes are a group of enzymes that hydrolyze pectin substances [3,4,5]. Based on the catalysis of pectin substrates, the pectinolytic enzymes can be classified into protopectinase, pectin methylesterases, and depolymerase (hydrolase and transeliminase) [3,6]. These enzymes are widely used in industrial processes, such as the retting and degumming of plant fibers, the fermentation of tea and coffee, clarification of fruit juices and wine, wastewater remediation, and animal feed production, which comprise 25% shares in the global food and beverage enzyme market [7,8].

Pectinolytic enzymes are widely distributed in bacteria, fungi, yeast, insects, nematodes, protozoans, and plants [1]. With the increase demand for pectinolytic enzymes, the discovery of new pectinolytic enzymes with proper characteristics is of great interest. Microorganisms represent an attractive and ideal resource for pectinolytic enzyme discovery [7,9]. In fact, multiple microbial pectinolytic enzymes have been identified from bacteria, fungi, and yeasts [7,10]. An alkaline pectinolytic enzyme was identified from *Bacillus subtilis* ZGL14 isolated from soil, and its optimal temperature and pH were 50 °C and 8.6, respectively. The purified enzyme showed strong thermo-stability and good alkali resistance [11]. The maximum pectinolytic activity of a new pectinolytic enzyme derived from *B. subtilis* Btk 27 was achieved at pH 7.5 and 50 °C, which has potential application in coffee processing [12]. An extracellular exo-polygalacturonase produced by *Penicillium notatum* displayed its optimal activity at pH 6.0 and 50 °C [5]. Most fungal pectinolytic enzymes are acidic, while alkaline pectinolytic enzymes are mainly secreted by bacteria [10,11,12,13]. Alkaline pectinolytic enzymes can be applied in the pretreatment of food processing wastewater, and fabric and paper production wastewater [13].

Efficient lignocellulose degradation microbiota often contains large amounts of lignocellulose-degrading enzymes, and many efficient pectinolytic enzymes and other carbohydrate-active enzymes (CAZy) are available. In the past few years, some pectinolytic enzymes derived from diverse environmental microbiota have been predicted or characterized. A novel pectate lyase with optimal activity at 40 °C and pH 11.0 was identified from the hot spring metagenome, which has promising application in pectin-removal processing [14]. A total of 1756 genes encoding putative pectinolytic enzymes were identified from an apple pomace-adapted compost microbiota, and 129 of them were novel. However, none of the pectinolytic genes were characterized [15]. The tobacco leaf fermentation microbiota harbored diverse lignocellulose-degrading enzymes, and some of them were pectinolytic enzymes [16]. More than 90,000 genes/fragments encoding for CAZy were identified in the pulp and paper wastewater treatment microbiota (PW), and some of them were pectinolytic enzymes [17]. Two efficient xylanases derived from this efficient lignocellulose degradation microbiota have been characterized, showing that lignocellulose-degrading enzymes with high-activity might be available in the microbiota [18].

In this study, we analyzed pectinolytic genes derived from the pulp and paper wastewater treatment microbiota, and full-length pectinolytic genes were predicted. Moreover, we cloned 23 potential pectinolytic genes assigned to the GH28 and PL1 family, and expressed them in *Escherichia coli.* The biochemical characterization of one GH28 pectinolytic enzyme, PW-pGH28-3, was further analyzed, and bioinformatic insight into this pectinolytic enzyme was implemented.

## 2. Materials and Methods

### 2.1. Strains, Plasmid, and Reagents

*E. coli* strains TOP10 and BL21(DE3) were bought from Tolo Biotech Co. Ltd. (Anhui, China). The FastPure^®^ Gel DNA Extraction Mini Kit and Fast Pure Plasmid Mini Kit were bought from Vazyme Biotech Co. Ltd. (Nanjing, China). The pectinolytic enzyme substrate, polygalacturonic acid, was bought from Sigma-Aldrich (Darmstadt, Germany). The 3,5-dinitrosalicylic acid (DNS) and other chemical reagents were bought from the China National Pharmaceutical Group Corporation (Beijing, China).

### 2.2. Screening of Novel Pectinolytic Genes and Phylogenetic Analysis

A set of CAZy genes (>240 pectinolytic genes/gene fragments) were recovered from the pulp and paper wastewater treatment microbiota [5,13]. A total of 100 annotated pectinolytic genes/gene fragments were predicted to be full-length. A total of 23 potential pectinolytic genes, including 14 GH28 family genes and 9 PL1 family genes, were selected for expression, as they were predicted to be full-length and had not been previously characterized. These 23 genes were aligned with some high-activity pectinolytic enzymes downloaded from the CAZy database. The phylogenetic tree of the 23 pectinolytic genes and some known pectinolytic genes were built with MEGA 11 [19]. The 100 predicted pectinolytic genes were submitted to GenBank with the accession numbers of OP326391-OP326461 and OP326468-OP326496.

### 2.3. Cloning of the Pectinolytic Genes

The 23 primer pairs for the selected pectinolytic genes were designed (Table S1) and used for amplification of the corresponding pectinolytic genes. These genes were ultimately verified by DNA sequencing, and they were named as PW-pGH28-1 to PW-pGH28-14 and PW-pPL-1 to PW-pPL-9, as they were derived from the pulp and paper wastewater treatment microbiota (PW) (Table 1). The cloned pectinolytic genes were inserted into the pET-28a (+) expression vector by the One Step Cloning Kit (Vazyme, Nanjing, China), separately. The 23 recombinant plasmids were individually transformed into the *E. coli* TOP10 strain. After verification, correct recombinant plasmids were further transformed into the *E. coli* BL21(DE3) strain for protein expression.

### 2.4. Expression of the Pectinolytic Genes

The *E. coli* BL21(DE3) strains with recombinant plasmid were inoculated into 100 mL LB medium with 50 µg/mL kanamycin in a 500 mL shake flask, and further cultivated at 37 °C and 200 rpm. When the OD600 value reached 0.6-0.8, IPTG was added to reach 100 µM, in order to induce pectinolytic enzyme expression. After cultivation for 6 h at 25 °C, cell cultures were harvested by centrifugation at 7000× *g* for 10 min. The harvested cell cultures were washed with phosphate buffered saline (PBS) buffer (pH 7.4) and then disrupted by supersonic waves (Xiaomei, Kunshan, China) on ice with 150 W for 15 min (3 s pulse and 5 s interval). Finally, the cell lysates were centrifuged at 12,000 rpm for 20 min, and the supernatants were used for pectinolytic activity evaluation.

Polygalacturonic acid was used as the pectinolytic enzyme substrate, and crude pectinolytic activity was determined by the DNS method [20]. A total of 30 µL appropriately diluted cell supernatants and 30 µL 1% polygalacturonic acid were incubated together at 20 °C for 20 min, 40 °C for 20 min, and 60 °C for a further 20 min. Subsequently, 60 µL DNS reagent was added to each tube to stop the enzymatic reaction, and the mixtures were incubated at 95 °C for 5 min. The crude activity of the expressed pectinolytic enzymes could be determined [21].

### 2.5. Expression and Purification of PW-pGH28-3 Pectinolytic Enzyme

The BL21 (DE3) strain harboring PW-pGH28-3 was incubated overnight in a shaker at 37 °C and 200 rpm. Then, 4 mL of the overnight culture was inoculated to 200 mL fresh LB medium (50 µg/mL kanamycin) in 1 L shake flask. When the OD600 reached 0.6–0.8, IPTG was added to reach 100 µM, and the cells were cultivated at 25 °C for another 6 h. Cells were harvested and washed three times with PBS, then suspended in 20 mL PBS with 1 mM phenylmethylsulfonyl fluoride (PMSF, protease inhibitor). The cells were disrupted by sonication. The cell lysates were centrifuged at 12,000 rpm at 4 °C for 20 min, and the supernatant was collected as crude enzyme solution. Ni NTA beads (Smart-Lifesciences, Jiangsu, China) were used for protein purification, and the purified PW-pGH28-3 protein was concentrated in 10 kDa ultrafiltration tubes (Merck Millipore, Darmstadt, Germany). The purity of PW-pGH28-3 was determined by sodium dodecyl sulfate-polyacrylamide gel electrophoresis (SDS-PAGE), and the concentration was measured with the Bradford method (Sangon Biotech, Shanghai, China).

### 2.6. Effects of Temperature and pH on PW-pGH28-3 Activity

The optimal temperature of PW-pGH28-3 was determined by incubating the reaction mixtures at 20 °C, 25 °C, 30 °C, 35 °C, 40 °C, 45 °C, 50 °C, 55 °C, 60 °C, and 65 °C, separately. A total of 1% polygalacturonic acid was dissolved in 10 mM Tris-HCl buffer (pH 7.4), the 100 µL reaction mixture consisted of 50 µL 1% polygalacturonic acid, and 50 µL of the appropriately diluted PW-pGH28-3 solution. After 10 min, 100 μL DNS reagent was added to terminate the reaction. Then, the absorbance at 540 nm was detected. The pectinolytic activity (U/mg protein) was defined as the amount of D-galacturonic acid released/min/mg enzyme. The data were reported based on three independent experiments. The highest pectinolytic activity detected was defined as 100%.

In order to investigate the optimal pH of PW-pGH28-3, the reaction temperature was fixed at 40 °C (the measured optimal temperature). Different buffers with a wide pH range were used including disodium phosphate-citrate buffer (pH 5.0–6.0), Tris-HCl buffer (pH 6.0–9.0), and glycine-NaOH buffer (pH 9.0-10.0). The reaction system, reaction time as well as the pectinolytic activity detection method were the same as the optimal temperature determination experiment.

### 2.7. Detecting the Hydrolysates of PW-pGH28-3 by Thin-Layer Chromatography (TLC)

The hydrolysates of PW-pGH28-3 were analyzed by TLC. First, 100 μL 1% polygalacturonic acid was mixed with 100 μL enzyme solution and reacted for 10 min under the optimal reaction condition (40 °C, pH 8.0). The reaction was stopped by placing the catalysis samples in a water bath at 95 °C for 5 min, and centrifuged at 12,000 *g* for 10 min. The enzymatic hydrolysates from the supernatant were used as the sample for TLC analysis. Silica gel GF254 plates (Qingdao Ocean Chemical Co. Ltd., Qingdao, China) were used as a stationary phase, and the mobile phase was chloroform:glacial acetic acid:water (6:7:1, by volume). The prepared samples were applied (3 μL) onto the TLC plates, and the plates were developed until the solvent was about 1 cm below the top of the plate. Then, the plates were dried at room temperature. The developed TLC plates were visualized by spraying the color developing agent (diphenylamine/aniline/phosphoric acid/acetone) uniformly, and then placed in an oven at 100 °C for 10 min until brown spots appeared [22].

### 2.8. Bioinformatics Analyses of PW-pGH28-3

The SignalP-6.0 server was used to predict the signal peptide of PW-pGH28-3. The SWISS-MODEL server was used to predict the 3D structure of PW-pGH28-3, and the modeling structure was visualized and aligned with other known enzyme structures by the PyMOL molecular visualization system.

## 3. Results

### 3.1. Insights into the Pectinolytic Genes Derived from Pulp and Paper Wastewater Treatment Microbiota

**Figure 1:**
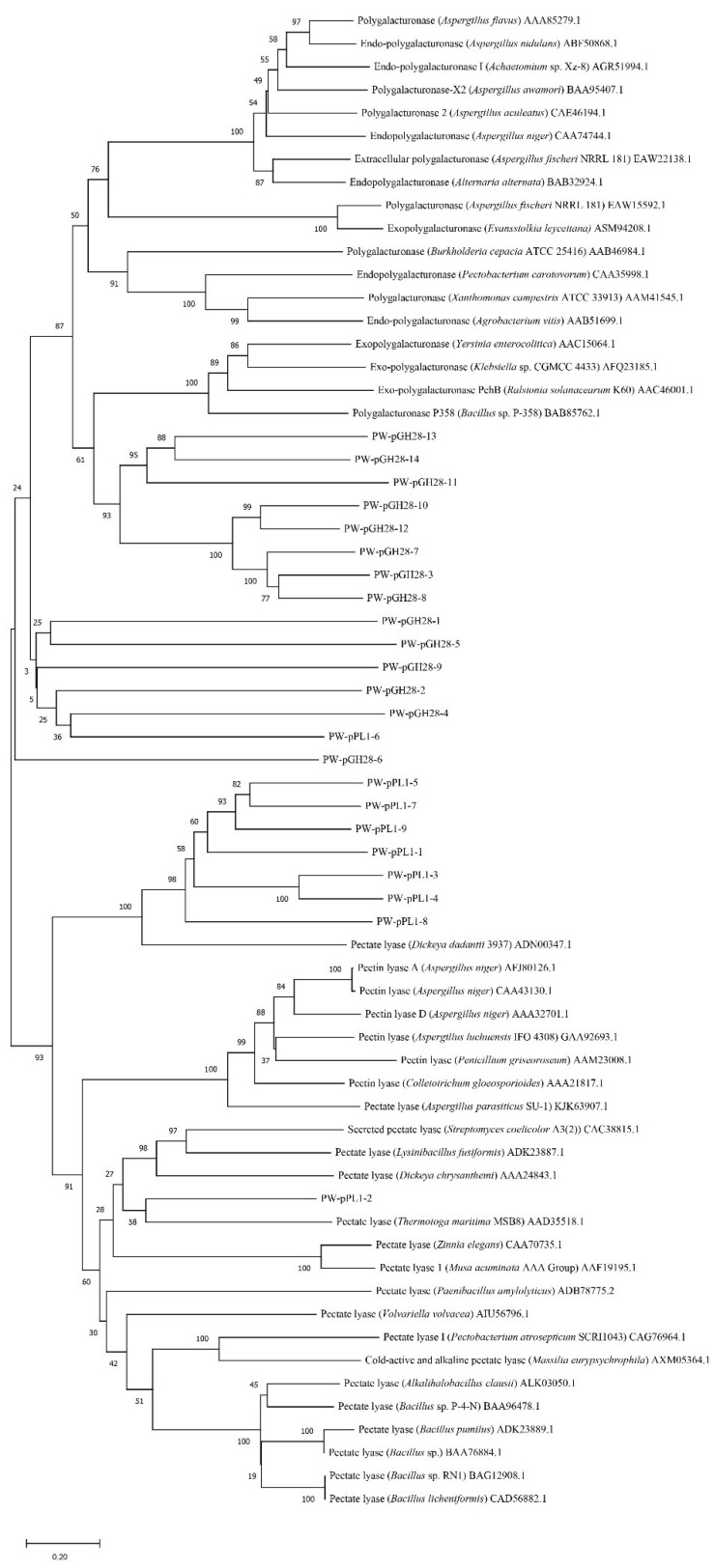
The phylogenetic tree of the 23 predicted pectinolytic genes and some known pectinolytic genes downloaded from the CAZy database. The tree was built with MEGA 11 based on amino acid sequence identity.

More than 200 pectinolytic genes/gene fragments assigned to the GH28 family and PL1 family were predicted in the pulp and paper wastewater treatment microbiota based on dbCAN [5,13,23]. Among them, 100 pectinolytic genes were predicted to be full-length (Figure S1 and Table S1). Based on the annotation and phylogenetic analysis, the genes that are similar with known pectinolytic genes or clustered with known pectinolytic genes were selected. A total of 23 predicted pectinolytic genes, including 14 from the GH28 family and 9 PL1 family genes, were cloned (Table 1). These pectinolytic gene fragment sizes ranged from 1347 bp to 2514 bp, and they showed 45.2% to 99.8% sequence identity with the previously known genes in the GenBank database. However, some of the predicted pectinolytic enzymes were different from the known pectinolytic enzymes (Figure 1). The crude enzyme activity results showed that 5 of the 23 pectinolytic genes had pectinolytic activity (Table 1 and Figure S2). Among them, PW-pGH28-3 showed a 58.4% sequence identity to an exo-polygalacturonase of *Aquipluma nitroreducens*, hinting that PW-pGH28-3 might be derived from the *Aquipluma* species. PW-pGH28-10 showed a 76.4% sequence identity to an exo-poly-alpha-D-galacturonosidase of *Bacteroidetes* bacterium. The crude pectinolytic activity of PW-pGH28-3 was high (Figure S2), therefore, PW-pGH28-3 was selected for further purification and characterization.

### 3.2. Purification and Characterization of PW-pGH28-3

**Figure 2:**
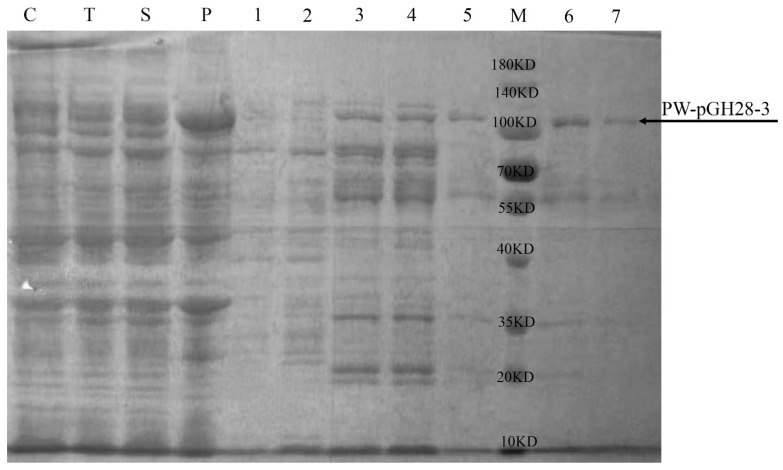
The purification of PW-pGH28-3. Lane C, protein supernatant of *E. coli* BL21; Lane T, total protein mixture of *E. coli* BL21 expressed PW-pGH28-3; Lane S, supernatant protein mixture of *E. coli* BL21 expressed PW-pGH28-3; Lane P, precipitated protein mixture of *E. coli* BL21 expressed PW-pGH28-3; Lane 1, protein washed with 20 mM imidazole buffer; Lane 2, protein washed with 50 mM imidazole buffer; Lane 3, protein washed with 100 mM imidazole buffer; Lane 4, protein washed with 300 mM imidazole buffer for the first time; Lane 5, protein washed with 300 mM imidazole buffer for the second time; Lane M, molecular mass standards; Lane 6, protein washed with 500 mM imidazole buffer for the first time; Lane 7, protein washed with 500 mM imidazole buffer for the second time.

**Figure 3:**
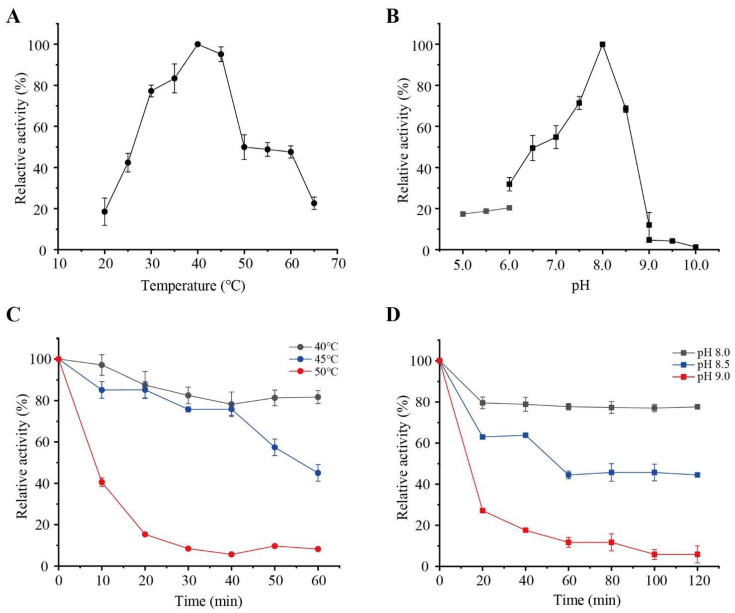
The enzymatic characteristics of PW-pGH28-3 derived from the pulp and paper wastewater treatment microbiota. (**A**) Measuring the optimal temperature of PW-pGH28-3. (**B**) Measuring the optimal pH of PW-pGH28-3. (**C**) Thermal stability of the PW-pGH28-3, and the relative pectinolytic activities detected at 40 °C, 45 °C, and 50 °C were shown. (**D**) pH stability of PW-pGH28-3, and the relative pectinolytic activities detected at pH 8.0, 8.5, and 9.0 were shown. The values represent the mean values of the triplicate experiments, and the error bar indicates the standard deviation.

The predicted molecular weight of PW-pGH28-3 was about 100 kDa, and the purified PW-pGH28-3 was close to 100 kDa (Figure 2). The optimal temperature and pH of PW-pGH28-3 were 40 °C and pH 8.0, respectively (Figure 3A,B). The pectinolytic activity of PW-pGH28-3 sustained >70% activity from 30 °C to 45 °C (Figure 3A); PW-pGH28-3 can sustain >80% activity when kept at 40 °C for 60 min and >80% activity at 45 °C for 40 min, respectively (Figure 3C). The pectinolytic activity of PW-pGH28-3 can sustain >60% activity from pH 7.5 to 8.5 (Figure 3B), and it can sustain >70% activity in pH 8.0 for 120 min (Figure 3D). The results suggest that PW-pGH28-3 is a stable alkaline pectinolytic enzyme that can work across a wide pH range. The pectinolytic activity of PW-pGH28-3 was 13.5 ± 1.1 U/mg protein at pH 8.0 and 40 °C.

### 3.3. Bioinformatics Analysis and Exo-Polygalacturonase Activity Confirmation of PW-pGH28-3

PW-pGH28-3 has 822 amino acids, which is larger than most of the reported pectinolytic enzymes including endo- and exo-polygalacturonases [24,25,26]. The SignalP 6.0 predicted that PW-pGH28-3 had a Sec/SPI signal peptide. The signal peptide cleavage site was between amino acids 22 and 23, and the probability value was 0.964 (Figure S3). As Sec/SPI is a secretory signal peptide, it suggests that PW-pGH28-3 is a secretory pectinolytic enzyme.

The optimal template, an exo-poly-alpha-D-galacturonosidase derived from *Thermotoga maritima* (Tm_ExoPG, PDB ID: 3JUR) [25], was used to predict the PW-pGH28-3 structure in SWISS-MODEL server. The sequence identity between PW-pGH28-3 and the template was 35.49%. The GMQE value of the model structure of PW-pGH28-3 was 0.43, and the QMEANDisCo Global value was 0.7 ± 0.05 (Figure S4).

**Figure 4:**
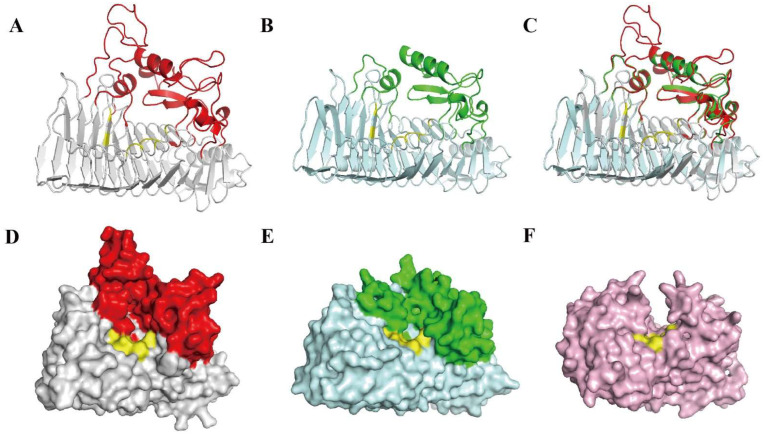
A structure comparison of PW-pGH28-3 and two typical pectinolytic enzymes of Tm_ExoPG and Ec_EndoPG. (**A**) The predicted structure of PW-pGH28-3. The right-handed parallel β-helix fold is marked with light-grey, and several turns and loops lining the active site cleft are marked with red. (**B**) The structure of Tm_ExoPG. The right-handed parallel β-helix fold is marked with light-cyan, and several turns and loops lining the active site cleft are marked with green. (**C**) Superimposition of the predicted PW-pGH28-3 and Tm_ExoPG. (**D**) The surface structure of PW-pGH28-3. (**E**) The surface structure of Tm_ExoPG. (**F**) The surface structure of Ec_EndoPG, which is marked in light-pink. Eight conserved residues in the active site of these three pectinolytic enzymes are all marked in yellow.

The modeling results show that the PW-pGH28-3 structure adopts a conventional right-handed parallel β-helix fold, and its active site cleft is open at one side (Figure 4A, D). Consequently, the cleft is only accessible from the C-terminal side of the β-helix, and displays a pocket-like active site (Figure 4D), suggesting that PW-pGH28-3 has an exo-pectinolytic enzyme activity. Therefore, PW-pGH28-3 might degrade pectin and generate carbohydrate products with a uniform degree of polymerization. Tm_ExoPG (Figure 4B, E) and the *Yersinia enterocolitica* exo-polygalacturonase (Ye_ExoPG, PDB ID: 2UVE) had similar structures [26], while the endo-polygalacturonases of *Erwinia carotovora* ssp. *Carotovora* (Ec_EndoPG, PDB ID: 1BHE) displayed a tunnel-like active site (Figure 4F) [26]. In several endo- and exo-polygalacturonases, eight amino acids were strictly conserved [25,26,27,28]. In the PW-pGH28-3 model structure, eight amino acids (N551, D553, D574, D575, H608, G609, R639, and K641) were located at similar positions of several endo- and exo-polygalacturonases (Figure S5). Among them, the three proposed catalytic aspartates (D553, D574, D575) were positioned at the bottom of the pocket (Figure 4D, marked in yellow). Thus, PW-pGH28-3 might be a novel exo-polygalacturonase. Residues 1–307 were missing at the N-terminal of the PW-pGH28-3 model structure, and this is similar to the Ye_ExoPG FN3 domain, which plays a possible alternative role in carbohydrate recognition [26].

Bioinformatic analysis suggested that PW-pGH28-3 might be an exo-polygalacturonase, and we tested its hydrolytic activity by TLC (Figure S5). PW-pGH28-3 is able to hydrolyze polygalacturonic acid into D-galacturonic acid (monose), which confirms the exo-activity of PW-pGH28-3, showing PW-pGH28-3 is a new exo-polygalacturonase.

## 4. Discussion

Metagenomics and other microbiome strategies have been applied to recover novel lignocellulose-degrading genes and other functional genes in environmental microbiota [20,29]. Millions of CAZy genes have been recovered from cow rumen, sheep rumen, and other efficient lignocellulose degradation microbiota [30,31]. Many of the cow rumen CAZy genes were rediscovered, showing that the metagenomic data already provided redundant information for lignocellulose-degrading gene recovery and the novelty of genes in the uncovered natural microbiota might be low [31,32]. Until now, only a few predicted lignocellulose-degrading enzymes or other CAZy genes have been characterized, thus, the actual functions of most genes are unknown and the function of the predicted genes could not be verified [33]. Therefore, further characterizing the predicted genes would give insights into the lignocellulose-degrading enzyme functions of the efficient lignocellulose degradation microbiota. Moreover, some efficient lignocellulose-degrading enzymes might be recovered and applied in the food, textile, and other related industries.

In our previous study, several hundred pectinolytic genes were predicted based on the dbCAN (version 6.0) database using HMMER 3.2, and they were parts of the huge amounts of CAZy genes identified from the pulp and paper wastewater treatment microbiota [17]. 40 xylanase genes were expressed, and 14 of the expressed enzymes (35.0%) showed xylanase activity [18]. 23 predicted pectinolytic genes were expressed, and only 5 of them (21.7%) showed pectinolytic gene activity in this study. The prediction of some pectinolytic genes was not accurate, which resulted in the low recovery of pectinolytic enzymes (21.7%) from the microbiota. This suggested that functional characterization of metagenomic recovered genes was essential and the parameters used to predict functional genes should be further optimized. In the future, optimizing efficient lignocellulose-degrading gene prediction strategy is necessary, and the integration of machine learning or other artificial intelligence would increase the discovery of efficient lignocellulose-degrading genes [34,35]. Furthermore, the expression of pectinolytic genes with efficient vectors and the addition of activation tags might increase the successful expression of pectinolytic genes [36].

The PW-pGH28-3 was predicted to have exo-pectinolytic activity, and this was verified by the analysis of its hydrolysates. The bioinformatic analysis suggested active catalytic sites and substrate binding pockets, showing the potential pectin degradation mechanism of PW-pGH28-3. The optimal pH and temperature of PW-pGH28-3 were 8.0 and 40 °C, respectively, and it was stable at wide pH and temperature ranges, hinting that this pectinolytic enzyme might be applied in the food and textile industries. However, the pectinolytic activity of PW-pGH28-3 is low (13.5 ± 1.1 U/mg protein), so future protein engineering of PW-pGH28-3 should be applied to enhance its activity. Moreover, other high-activity pectinolytic enzymes might be available in the efficient lignocellulose degradation microbiota. As the efficient lignocellulose degradation microbiota often harbor large amounts of pectinolytic enzymes and other lignocellulose-degrading enzymes, developing a high-throughput lignocellulose-degrading enzyme characterization strategy, including gene prediction, gene expression, the lignocellulose-degrading enzyme activity test, and structure and catalytic mechanism analyses, is of great interest [35].

Lignocellulose is the most abundant biomass in nature, and it can generate functional sugars and other prebiotics. If the lignocellulose was not properly treated, it might become environment pollutants. Thus, the efficient treatment of lignocellulose wastes with microbiota can remove lignocellulose; meanwhile, methane and other biofuels can be generated [37]. The treatment of lignocellulose with enzymes costs high [38]. Nowadays, engineering microbes with efficient lignocellulose-degrading enzymes can increase their lignocellulose utilization ability. The engineered *Cordyceps militaris* can efficiently convert spent mushroom substrate to the high-value anticancer drug pentostatin, which paves the way for the future sustainable utilization of lignocellulose while producing high-value products [39]. With the development of synthetic biology, incorporating lignocellulose-degrading genes in engineered yeasts or other microbes might produce super microbial cell factories with the ability to use lignocellulose for high-value natural product biosynthesis [40].

## 5. Conclusions

In summary, we analyzed the predicted full-length pectinolytic genes in one efficient lignocellulose degradation microbiota derived from the pulp and paper wastewater treatment microbiota. 23 full-length genes were expressed, and 5 of them have pectinolytic activity. Further characterization provided insights into one pectinolytic enzyme of PW-pGH28-3. This study suggests that prediction methods for lignocellulose-degrading enzymes or other CAZy should be further improved and the experimental characterization of novel enzymes is essential for efficient enzyme screening and industrial application. In the future, cutting-edge technologies should be applied in characterizing and screening novel pectinolytic enzymes and other industrial enzymes.

## Figures and Tables

**Table 1:** The 23 pectinolytic genes derived from the pulp and paper wastewater treatment microbiota and their closest genes in the GenBank database.

Gene Name	Closest Genes	Accession Numbers	Sequence Identity	Endo- or Exo-Polygalacturonase Activity
PW-pGH28-1	Hypothetical protein [*Chloroflexi* bacterium]	NLG99247.1	45.2%	
PW-pGH28-2	Hypothetical protein [*Dehalococcoidia* bacterium]	NLE96346.1	69.9%	
PW-pGH28-3	Exo- polygalacturonase [*Aquipluma nitroreducens*]	BBE19954.1	58.4%	✓
PW-pGH28-4	Right-handed parallel beta-helix repeat-containing protein [*Methanobacterium* sp.]	MBI4813419.1	66.9%	
PW-pGH28-5	Right-handed parallel beta-helix repeat-containing protein [*Lentisphaerae* bacterium]	NLC83012.1	94.8%	
PW-pGH28-6	S-layer homology domain-containing protein [*Armatimonadetes* bacterium]	MBN1461142.1	49.5%	
PW-pGH28-7	Glycoside hydrolase family 28 protein [*Bacteroidales* bacterium]	NLX28859.1	99.8%	
PW-pGH28-8	Glycoside hydrolase family 28 protein [Bacteroidales bacterium]	MBN1107786.1	80.1%	✓
PW-pGH28-9	Disaggregatase related [*Methanomethylovorans* sp. PtaU1.Bin073]	OPY19182.1	79.0%	
PW-pGH28-10	Exo-poly-alpha-D-galacturonosidase [*Bacteroidetes* bacterium]	MBP1676534.1	76.4%	✓
PW-pGH28-11	Right-handed parallel beta-helix repeat-containing protein [*Bacteroidales* bacterium]	MBG0861178.1	81.0%	✓
PW-pGH28-12	Glycoside hydrolase family 28 protein [*Paludibacter* sp.]	MBP6662256.1	72.1%	
PW-pGH28-13	TPA: hypothetical protein [*Chloroflexi* bacterium]	HFI27235.1	54.8%	
PW-pGH28-14	Glycoside hydrolase family 28 protein [*Bacteroidales* bacterium]	MBK7713019.1	57.4%	
PW-pPL1-1	T9SS type A sorting domain-containing protein [*Catalinimonas alkaloidigena*]	WP_089687757.1	52.1%	
PW-pPL1-2	Pectate trisaccharide-lyase precursor [*Bacteroidetes* bacterium A Durb.BinA395]	OPZ03270.1	73.6%	✓
PW-pPL1-3	Polysaccharide lyase [*Bacteroides* sp. 51]	WP_163173617.1	82.6%	
PW-pPL1-4	Polysaccharide lyase [*Paludibacter* sp. SCN 50-10]	ODT57447.1	83.9%	
PW-pPL1-5	Hypothetical protein BGP01_06875 [*Paludibacter* sp. 47-17]	OJX91998.1	57.5%	
PW-pPL1-6	Pectate lyase [*Phycisphaerae* bacterium]	MBN2560505.1	80.7%	
PW-pPL1-7	Hypothetical protein [*Bacteroidetes* bacterium]	MBP1676284.1	61.5%	
PW-pPL1-8	Pectate lyase precursor [*Deltaproteobacteria* bacterium ]	MBM4340551.1	69.6%	
PW-pPL1-9	Pectate lyase [*Bacteroidales* bacterium]	NLD63827.1	80.6%	

## Data Availability

Not Applicable.

## Supplementary Materials

The following supporting information can be downloaded at: https://www.mdpi.com/article/10.3390/biom12101388/s1, Figure S1: The phylogenetic tree of the 100 pectinolytic genes predicted from the pulp and paper wastewater treatment microbiota. Figure S2: The crude pectinolytic activity of the selected 23 expressed pectinolytic enzymes. Figure S3: Signal peptide prediction of PW-pGH28-3 by SignalP 6.0 server. The predicted cleavage site was shown. Figure S4: Evaluation of PW-pGH28-3 model by SWISS-MODEL server. Figure S5: Sequence alignment of PW-pGH28-3 and three other known pectinases. Figure S6: The hydrolysates of polygalacturonic acid catalyzed by PW-pGH28-3. Table S1: The sequence identity of the 100 predicted full-length pectinolytic genes derived from pulp and paper wastewater treatment microbiota to their most similar genes and their accession numbers in GenBank database. Table S2: 23 pairs of primers used to amplify the 23 pectinolytic genes derived from pulp and paper wastewater treatment microbiota.

## References

[1] Shrestha S., Kognou A.L.M., Zhang J., Qin W. (2021). Different facets of lignocellulosic biomass including pectin and its perspectives. Waste Biomass Valori..

[2] Picot-Allain M.C.N., Ramasawmy B., Emmambux M.N. (2022). Extraction, characterisation, and application of pectin from tropical and sub-tropical fruits: A Review. Food Rev. Int..

[3] Jayani R.S., Saxena S., Gupta R. (2005). Microbial pectinolytic enzymes: A review. Process Biochem..

[4] Amin F., Bhatti H.N., Bilal M., Asgher M. (2017). Multiple parameter optimizations for enhanced biosynthesis of exo-polygalacturonase enzyme and its application in fruit juice clarification. Int. J. Food Eng..

[5] Amin F., Bhatti H.N., Bilal M., Asgher M. (2017). Purification, kinetic, and thermodynamic characteristics of an exo-polygalacturonase from penicillium notatum with industrial perspective. Appl. Biochem. Biotechnol..

[6] Shrestha S., Rahman M.S., Qin W. (2021). New insights in pectinase production development and industrial applications. Appl. Microbiol. Biotechnol..

[7] Amin F., Bhatti H.N., Bilal M. (2019). Recent advances in the production strategies of microbial pectinases—A review. Int. J. Biol. Macromol..

[8] Kashyap D.R., Vohra P.K., Chopra S., Tewari R. (2001). Applications of pectinases in the commercial sector: A review. Bioresour. Technol..

[9] Garg G., Singh A., Kaur A., Singh R., Kaur J., Mahajan R. (2016). Microbial pectinases: An ecofriendly tool of nature for industries. 3 Biotech.

[10] Kavuthodi B., Sebastian D. (2018). Review on bacterial production of alkaline pectinase with special emphasis on *Bacillus* species. Biosci. Biotechnol. Res. Comm..

[11] Yu P., Zhang Y., Gu D. (2017). Production optimization of a heat-tolerant alkaline pectinase from *Bacillus subtilis* ZGL14 and its purification and characterization. Bioengineered.

[12] Oumer O.J., Abate D. (2017). Characterization of pectinase from *Bacillus subtilis* strain Btk 27 and its potential application in removal of mucilage from coffee beans. Enzyme Res..

[13] Hoondal G.S., Tiwari R.P., Tewari R., Dahiya N., Beg Q.K. (2002). Microbial alkaline pectinases and their industrial applications: A review. Appl. Microbiol. Biotechnol..

[14] Sharma N., Sahoo D., Rai A.K., Singh S.P. (2022). A highly alkaline pectate lyase from the Himalayan hot spring metagenome and its bioscouring applications. Process Biochem..

[15] Zhou M., Guo P., Wang T., Gao L., Yin H., Cai C., Gu J., Lü X. (2017). Metagenomic mining pectinolytic microbes and enzymes from an apple pomace-adapted compost microbial community. Biotechnol. Biofuels.

[16] Tao J., Chen Q., Chen S., Lu P., Chen Y., Jin J., Li J., Xu Y., He W., Long T. (2022). Metagenomic insight into the microbial degradation of organic compounds in fermented plant leaves. Environ. Res..

[17] Liang J., Mai W., Wang J., Li X., Su M., Du J., Wu Y., Dai J., Tang Q., Gao J. (2021). Performance and microbial communities of a novel integrated industrial-scale pulp and paper wastewater treatment plant. J. Clean Prod..

[18] Wang J., Liang J., Li Y., Tian L., Wei Y. (2021). Characterization of efficient xylanases from industrial-scale pulp and paper wastewater treatment microbiota. AMB Express.

[19] Kumar S., Stecher G., Tamura K. (2016). MEGA7: Molecular evolutionary genetics analysis version 7.0 for bigger datasets. Mol. Biol. Evol..

[20] Wei Y., Zhou H., Zhang J., Zhang L., Geng A., Liu F., Zhao G., Wang S., Zhou Z., Yan X. (2015). Insight into dominant cellulolytic bacteria from two biogas digesters and their glycoside hydrolase genes. PLoS ONE.

[21] Yan X., Geng A., Zhang J., Wei Y., Zhang L., Qian C., Wang Q., Wang S., Zhou Z. (2013). Discovery of (hemi-) cellulase genes in a metagenomic library from a biogas digester using 454 pyrosequencing. Appl. Microbiol. Biotechnol..

[22] Yan J., Guo X., Li X., Wu X., Gou X. (2006). TLC to fleetly analyze monosaccharide composition of polysaccharide. Food Sci..

[23] Zhang H., Yohe T., Huang L., Entwistle S., Wu P., Yang Z., Busk P.K., Xu Y., Yin Y. (2018). dbCAN2: A meta server for automated carbohydrate-active enzyme annotation. Nucleic Acids Res..

[24] Bonivento D., Pontiggia D., Matteo A.D., Fernandez-Recio J., Salvi G., Tsernoglou D., Cervone F., Lorenzo G.D., Federici L. (2008). Crystal structure of the endopolygalacturonase from the phytopathogenic fungus *Colletotrichum lupini* and its interaction with polygalacturonase-inhibiting proteins. Proteins.

[25] Pijning T., van Pouderoyen G., Kluskens L., van der Oost J., Dijkstra B.W. (2009). The crystal structure of a hyperthermoactive exopolygalacturonase from *Thermotoga maritima* reveals a unique tetramer. FEBS Lett..

[26] Abbott D.W., Boraston A.B. (2007). The structural basis for exopolygalacturonase activity in a family 28 glycoside hydrolase. J. Mol. Biol..

[27] Pickersgill R., Smith D., Worboys K., Jenkins J. (1998). Crystal structure of polygalacturonase from *Erwinia carotovora* ssp. *carotovora*. J. Biol. Chem..

[28] Kester H.C., Kusters-van Someren M.A., Müller Y., Visser J. (1996). Primary structure and characterization of an exopolygalacturonase from *Aspergillus tubingensis*. Eur. J. Biochem..

[29] Liu N., Li H., Chevrette M.G., Zhang L., Cao L., Zhou H., Zhou X., Zhou Z., Pope P.B., Currie C.R. (2019). Functional metagenomics reveals abundant polysaccharide-degrading gene clusters and cellobiose utilization pathways within gut microbiota of a wood-feeding higher termite. ISME J..

[30] Gharechahi J., Vahidi M.F., Bahram M., Han J., Ding X., Salekdeh G.H. (2021). Metagenomic analysis reveals a dynamic microbiome with diversified adaptive functions to utilize high lignocellulosic forages in the cattle rumen. ISME J..

[31] Stewart R.D., Auffret M.D., Warr A., Walker A.W., Roehe R., Watson M. (2019). Compendium of 4941 rumen metagenome-assembled genomes for rumen microbiome biology and enzyme discovery. Nat. Biotechnol..

[32] Hess M., Sczyrba A., Egan R., Kim T.-W., Chokhawala H., Schroth G., Luo S., Clark D.S., Chen F., Zhang T. (2011). Metagenomic discovery of biomass-degrading genes and genomes from cow rumen. Science.

[33] Batista-García R.A., del Rayo Sánchez-Carbente M., Talia P., Jackson S.A., O’Leary N.D., Dobson A.D., Folch-Mallol J.L. (2016). From lignocellulosic metagenomes to lignocellulolytic genes: Trends, challenges and future prospects. Biofuels Bioprod. Biorefining.

[34] Neves A.L., Yu J., Suzuki Y., Baez-Magana M., Arutyunova E., O’Hara E., McAllister T., Ominski K.H., Lemieux M.J., Guan L.L. (2021). Accelerated discovery of novel glycoside hydrolases using targeted functional profiling and selective pressure on the rumen microbiome. Microbiome.

[35] Foroozandeh Shahraki M., Farhadyar K., Kavousi K., Azarabad M.H., Boroomand A., Ariaeenejad S., Hosseini Salekdeh G. (2021). A generalized machine-learning aided method for targeted identification of industrial enzymes from metagenome: A xylanase temperature dependence case study. Biotechnol. Bioeng..

[36] Xiao C., Somerville C., Anderson C.T. (2014). POLYGALACTURONASE INVOLVED IN EXPANSION1 functions in cell elongation and flower development in Arabidopsis. Plant Cell.

[37] Basak B., Ahn Y., Kumar R., Hwang J.H., Kim K.H., Jeon B.H. (2022). Lignocellulolytic microbiomes for augmenting lignocellulose degradation in anaerobic digestion. Trends Microbiol..

[38] Del Pozo M.V., Fernández-Arrojo L., Gil-Martínez J., Montesinos A., Chernikova T.N., Nechitaylo T.Y., Waliszek A., Tortajada M., Rojas A., Huws S.A. (2012). Microbial β-glucosidases from cow rumen metagenome enhance the saccharification of lignocellulose in combination with commercial cellulase cocktail. Biotechnol. Biofuels.

[39] Zou G., Li B., Wang Y., Yin X., Gong M., Shang J., Wei Y., Li X., Bao D. (2021). Efficient conversion of spent mushroom substrate into a high value-added anticancer drug pentostatin with engineered Cordyceps militaris. Green Chem..

[40] Liu G., Qu Y. (2019). Engineering of filamentous fungi for efficient conversion of lignocellulose: Tools, recent advances and prospects. Biotechnol. adv..

