# Supplementary material for: Characterization of Novel Pectinolytic Enzymes Derived from the Efficient Lignocellulose Degradation Microbiota
Source: Biomolecules. 2022 Sep 29;12(10):1388. doi: 10.3390/biom12101388 (PMC9599418; doi:10.3390/biom12101388)
Supplement: Supplementary file 1 [file biomolecules-12-01388-s001.zip › biomolecules-1912753-supplementary.pdf]

Supplementary Information

Characterization of Novel Pectinolytic Enzymes Derived from the Efficient Lignocellulose Degradation Microbiota

Qin Miao [1,†], Xiaoling Zhang [1,†], Yitong Wang [1], Xiaoqi Li [1], Zheng Wang [2], Lingmin Tian [3], Lingbo Qu [1,4] and Yongjun Wei [1,5,*]

1 Laboratory of Synthetic Biology, School of Pharmaceutical Sciences, Zhengzhou University, Zhengzhou 450001, China
2 College of Life Science and Technology, Beijing University of Chemical Technology, Beijing 100029, China
3 Department of Food Science and Engineering, Jinan University, Guangzhou 510632, China
4 College of Chemistry, Zhengzhou University, Zhengzhou 450001, China
5 Jiangsu Collaborative Innovation Center of Chinese Medicinal Resources Industrialization, Nanjing University of Chinese Medicine, Nanjing 210023, China
* Correspondence: yongjunwei@zzu.edu.cn
† These authors contributed equally to this work.

Table of Contents

Figure S1. The phylogenetic tree of the 100 pectinolytic genes predicted from the pulp and paper wastewater treatment microbiota.

Figure S2. The crude pectinolytic activity of the selected 23 expressed pectinolytic enzymes. 30 μL appropriately diluted cell supernatants containing the 23 expressed pectinolytic enzymes and 30 μL 1% polygalacturonic acid were incubated together at 20°C for 20 min, 40°C for 20 min, and 60°C for another 20 min, respectively. Subsequently, 60 μL DNS reagent was added to each tube to stop the enzymatic reaction, and the mixtures were incubated at 95°C for 5 min. The crude pectinolytic activities of the enzymes were indicated in the figure.

Figure S3. Signal peptide prediction of PW-pGH28-3 by SignalP 6.0 server. The predicted cleavage site was shown.

Figure S4. Evaluation of PW-pGH28-3 model by SWISS-MODEL server. (A) The model details used to build PW-pGH28-3. (B) The QMEAN Z-Scores of the built model of PW-pGH28-3. (C) Comparison non-redundant set of PDB Structure with PW-pGH28-3. (D) Local quality estimate of the built model of PW-pGH28-3. The residues near 430-500 and 675-705 have low-value of predicted local similarity to target, which formed the coil structure. The residues of 320-429, 501-674, and 706-760 show high-value of predicted local similarity to target, which constituted the right-handed parallel β-helix fold of PW-pGH28-3 model. (E) The confidence of the 8 predicted conserved amino acid residues.

Figure S5. Sequence alignment of PW-pGH28-3 and three other known pectinases. Tm_ExoPG: an exo-poly-alpha-D-galacturonosidase derived from *Thermotoga maritima* (PDB ID: 3JUR); Ec_EndoPG: an endo-polygalacturonase of *Erwinia carotovora* ssp. *Carotovora* (PDB ID: 1BHE); Ye_ ExoPG: a *Yersinia enterocolitica* exo-polygalacturonase (PDB ID: 2UVE). The black boxes and triangles indicate 8 conserved amino acid residues which are present in all the 4 sequences.

Figure S6. The hydrolysates of polygalacturonic acid catalyzed by PW-pGH28-3. Line 1: D-galacturonic acid (positive control). Line 2: the hydrolysates of polygalacturonic acid catalyzed by PW-pGH28-3. Line 3: the hydrolysates of polygalacturonic acid catalyzed by inactivated PW-pGH28-3. Line 4: polygalacturonic acid (negative control).

Table S1. The sequence identity of the 100 predicted full-length pectinolytic genes derived from pulp and paper wastewater treatment microbiota to their most similar genes and their accession numbers in GenBank database.

Table S2. 23 pairs of primers used to amplify the 23 pectinolytic genes derived from pulp and paper wastewater treatment microbiota.

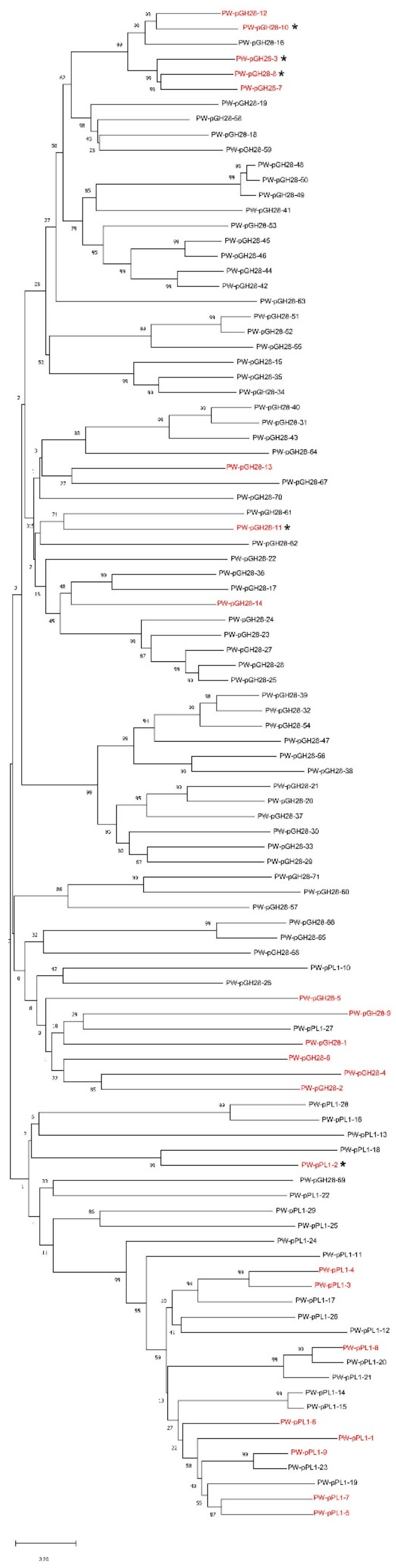
PW-pGH28-12
PW-pGH28-10 *
PW-pGH28-16
PW-pGH28-3 *
PW-pGH28-8 *
PW-pGH28-7
PW-pGH28-19
PW-pGH28-58
PW-pGH28-18
PW-pGH28-59
PW-pGH28-48
PW-pGH28-50
PW-pGH28-49
PW-pGH28-41
PW-pGH28-53
PW-pGH28-45
PW-pGH28-46
PW-pGH28-44
PW-pGH28-42
PW-pGH28-63
PW-pGH28-51
PW-pGH28-52
PW-pGH28-55
PW-pGH28-15
PW-pGH28-35
PW-pGH28-34
PW-pGH28-40
PW-pGH28-31
PW-pGH28-43
PW-pGH28-64
PW-pGH28-13
PW-pGH28-67
PW-pGH28-70
PW-pGH28-61
PW-pGH28-11 *
PW-pGH28-62
PW-pGH28-22
PW-pGH28-36
PW-pGH28-17
PW-pGH28-14
PW-pGH28-24
PW-pGH28-23
PW-pGH28-27
PW-pGH28-28
PW-pGH28-25
PW-pGH28-39
PW-pGH28-32
PW-pGH28-54
PW-pGH28-47
PW-pGH28-56
PW-pGH28-38
PW-pGH28-21
PW-pGH28-20
PW-pGH28-37
PW-pGH28-30
PW-pGH28-33
PW-pGH28-29
PW-pGH28-71
PW-pGH28-60
PW-pGH28-57
PW-pGH28-66
PW-pGH28-65
PW-pGH28-68
PW-pPL1-10
PW-pGH28-26
PW-pGH28-5
PW-pGH28-9
PW-pPL1-27
PW-pGH28-1
PW-pGH28-6
PW-pGH28-4
PW-pGH28-2
PW-pPL1-28
PW-pPL1-16
PW-pPL1-13
PW-pPL1-18
PW-pPL1-2 *
PW-pGH28-69
PW-pPL1-22
PW-pPL1-29
PW-pPL1-25
PW-pPL1-24
PW-pPL1-11
PW-pPL1-4
PW-pPL1-3
PW-pPL1-17
PW-pPL1-26
PW-pPL1-12
PW-pPL1-8
PW-pPL1-20
PW-pPL1-21
PW-pPL1-14
PW-pPL1-15
PW-pPL1-6
PW-pPL1-1
PW-pPL1-9
PW-pPL1-23
PW-pPL1-19
PW-pPL1-7
PW-pPL1-5

Figure S1. The phylogenetic tree of the 100 pectinolytic genes predicted from the pulp and paper wastewater treatment microbiota. The sequences were aligned, and the tree was constructed using neighbor-joining method with bootstrap method of 500. The selected 23 pectinolytic genes were colored with red, and the 5 expressed pectinolytic genes with activity were marker with asterisk (*).

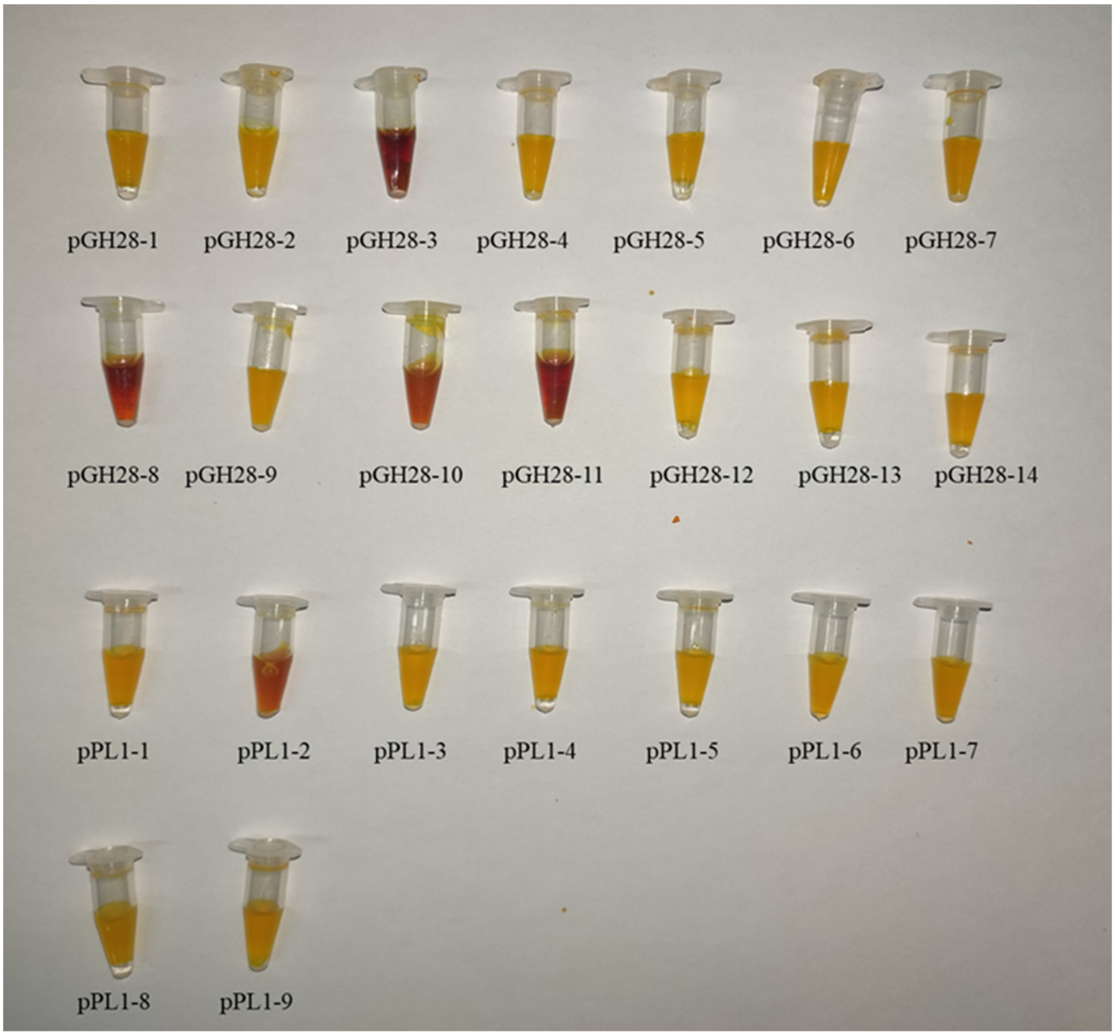

Figure S2. The crude pectinolytic activity of the selected 23 expressed pectinolytic enzymes. 30 μL appropriately diluted cell supernatants containing the 23 expressed pectinolytic enzymes and 30 μL 1% polygalacturonic acid were incubated together at 20°C for 20 min, 40°C for 20 min, and 60°C for another 20 min, respectively. Subsequently, 60 μL DNS reagent was added to each tube to stop the enzymatic reaction, and the mixtures were incubated at 95°C for 5 min. The crude pectinolytic activities of the enzymes were indicated in the figure.

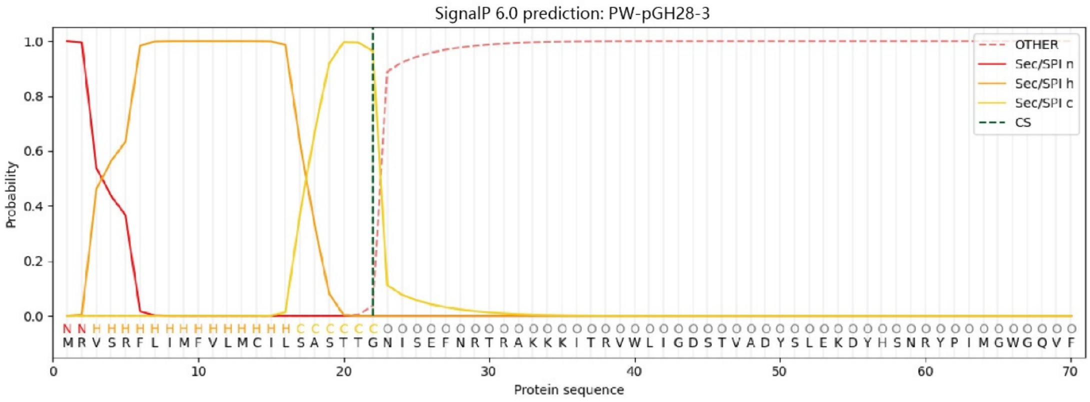

Figure S3. Signal peptide prediction of PW-pGH28-3 by SignalP 6.0 server. The predicted cleavage site was shown.

A

Model #01	File	Built with	Oligo-State	Ligands	GMQE	QMEANDisCo Global
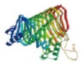	PDB	ProMod3 3.2.1	monomer (matching prediction)	None	0.43	0.69 ± 0.05

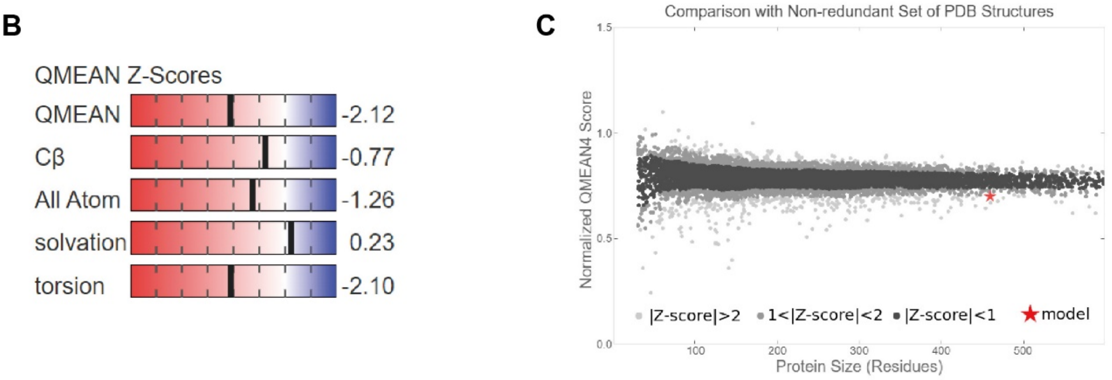

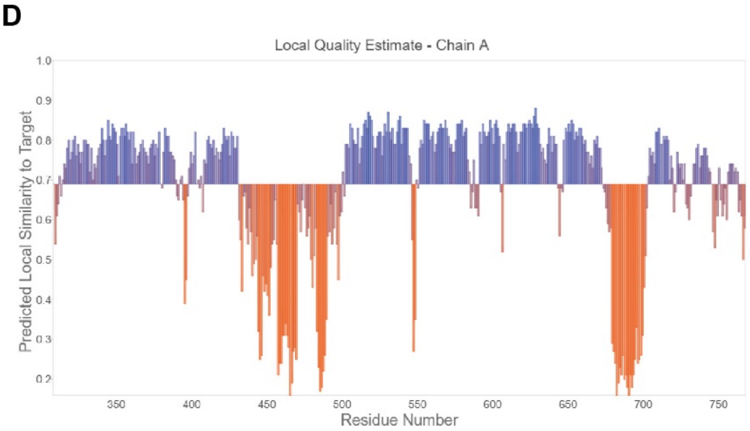

E

Conserved residues	Confidence (0~1)
N551	0.78
D553	0.79
D574	0.77
D575	0.80
H608	0.75
G609	0.82
R639	0.77
K641	0.79

Figure S4. Evaluation of PW-pGH28-3 model by SWISS-MODEL server. (A) The model details used to build PW-pGH28-3. (B) The QMEAN Z-Scores of the built model of PW-pGH28-3. (C) Comparison non-redundant set of PDB Structure with PW-pGH28-3. (D) Local quality estimate of the built model of PW-pGH28-3. The residues near 430-500 and 675-705 have low-value of predicted local similarity to target, which formed the coil structure. The residues of 320-429, 501-674, and 706-760 show high-value of predicted local similarity to target, which constituted the right-handed parallel β-helix fold of PW-pGH28-3 model. (E) The confidence of the 8 predicted conserved amino acid residues.

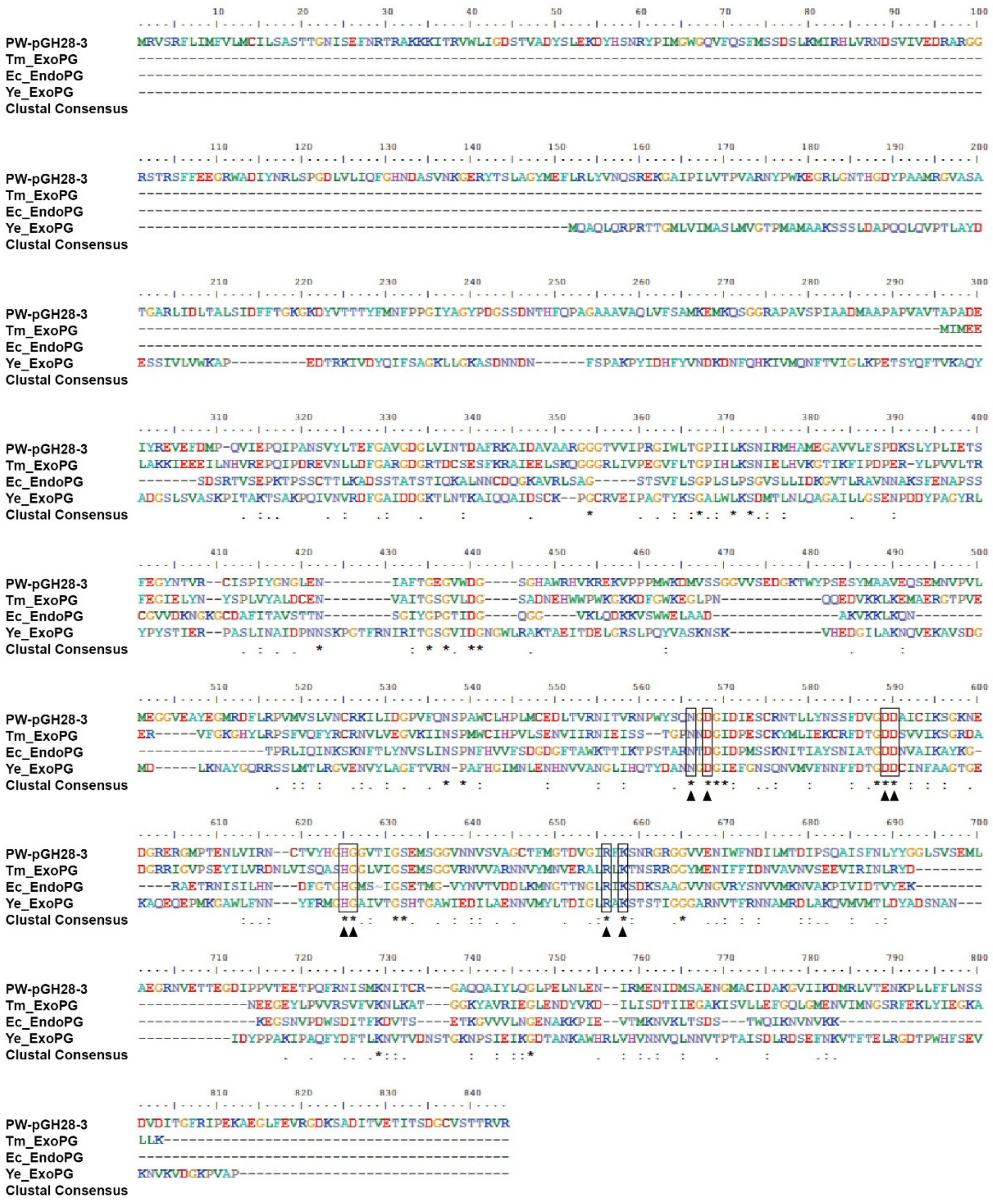

Figure S5. Sequence alignment of PW-pGH28-3 and three other known pectinases. Tm_ExoPG: an exo-poly-alpha-D-galacturonosidase derived from *Thermotoga maritima* (PDB ID: 3JUR); Ec_EndoPG: an endo-polygalacturonase of *Erwinia carotovora* ssp. *Carotovora* (PDB ID: 1BHE); Ye_ ExoPG: a *Yersinia enterocolitica* exo-polygalacturonase (PDB ID: 2UVE). The black boxes and triangles indicate 8 conserved amino acid residues which are present in all the 4 sequences.

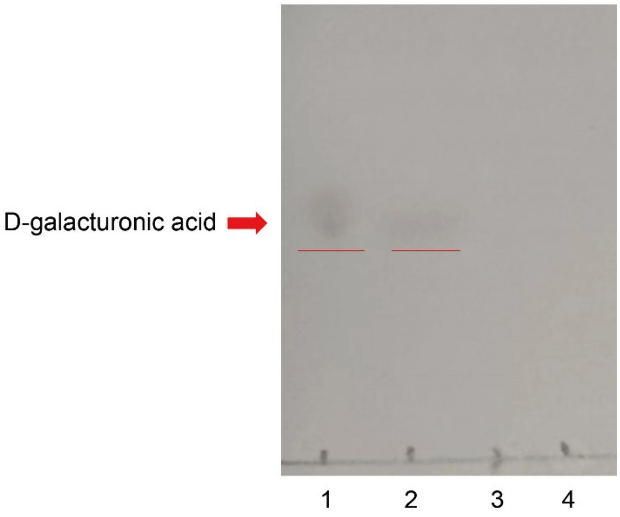

Figure S6. The hydrolysates of polygalacturonic acid catalyzed by PW-pGH28-3. Line 1: D-galacturonic acid (positive control). Line 2: the hydrolysates of polygalacturonic acid catalyzed by PW-pGH28-3. Line 3: the hydrolysates of polygalacturonic acid catalyzed by inactivated PW-pGH28-3. Line 4: polygalacturonic acid (negative control).

Table S1. The sequence identity of the 100 predicted full-length pectinolytic genes derived from pulp and paper wastewater treatment microbiota to their most similar genes and their accession numbers in GenBank database.

Gene name	Genbank numbers	The most similar genes [Microbial name]	Identity	Accession numbers
PW-pGH28-1	OP326391	Hypothetical protein [*Chloroflexi* bacterium]	45.2%	NLG99247.1
PW-pGH28-2	OP326392	Hypothetical protein [*Chloroflexi* bacterium]	45.2%	NLG99247.1
PW-pGH28-4	OP326394	Right-handed parallel beta-helix repeat-containing protein [*Methanobacterium* sp.]	66.9%	MBI4813419.1
PW-pGH28-5	OP326395	Right-handed parallel beta-helix repeat-containing protein [*Lentisphaerae* bacterium]	94.8%	NLC83012.1
PW-pGH28-6	OP326396	S-layer homology domain-containing protein [*Armatimonadetes* bacterium]	49.5%	MBN1461142.1
PW-pGH28-7	OP326397	Glycoside hydrolase family 28 protein [*Bacteroidales* bacterium]	99.8%	NLX28859.1
PW-pGH28-8	OP326398	Glycoside hydrolase family 28 protein [*Bacteroidales* bacterium]	80.1%	MBN1107786.1
PW-pGH28-9	OP326399	Disaggregatase related [*Methanomethylovorans* sp. PtaU1.Bin073]	79.0%	OPY19182.1
PW-pGH28-11	OP326401	Right-handed parallel beta-helix repeat-containing protein [*Bacteroidales* bacterium]	81.0%	MBG0861178.1
PW-pGH28-13	OP326403	Cellulase family glycosylhydrolase [*Aquisphaera* sp. JC669]	40.4%	WP_165225729.1
PW-pGH28-14	OP326404	Glycoside hydrolase family 28 protein [*Prolixibacteraceae* bacterium]	61.6%	MCK9412582.1
PW-pGH28-26	OP326416	Glycoside hydrolase family 28 protein [*Phycisphaerae* bacterium]	92.2%	NLZ04897.1
PW-pGH28-30	OP326420	Hypothetical protein [*Phycisphaerae* bacterium]	90.5%	MBP7050016.1
PW-pGH28-31	OP326421	Glycoside hydrolase [*Phycisphaerae* bacterium]	92.6%	NLZ05778.1
PW-pGH28-37	OP326427	Hypothetical protein [*Bacteroidetes* bacterium]	54.0%	MCP4313093.1
PW-pGH28-33	OP326423	Hypothetical protein [*Phycisphaerae* bacterium]	90.0%	NLZ05184.1
PW-pGH28-40	OP326430	Right-handed parallel beta-helix repeat-containing protein [*Phycisphaerae* bacterium]	90.4%	MBP7051897.1
PW-pGH28-43	OP326433	Right-handed parallel beta-helix repeat-containing protein [*Thermoguttaceae* bacterium]	93.9%	MCL4195699.1
PW-pPL1-11	OP326478	Por secretion system protein [*Paludibacter* sp. 47-17]	62.2%	OJX89770.1
PW-pGH28-60	OP326450	Hypothetical protein [*Planctomycetaceae* bacterium]	93.8%	NLS97349.1
PW-pGH28-61	OP326451	Right-handed parallel beta-helix repeat-containing protein [*Kiritimatiellae* bacterium]	90.9%	MBP8802606.1
PW-pGH28-62	OP326452	Right-handed parallel beta-helix repeat-containing protein [*Planctomycetes* bacterium]	61.3%	NRA37571.1
PW-pGH28-64	OP326454	TPA: hypothetical protein [*Bacteroidales* bacterium]	64.0%	HBZ20744.1
PW-pGH28-65	OP326455	Right-handed parallel beta-helix repeat-containing protein [*Anaerolineae* bacterium]	67.4%	MCD6284988.1
PW-pGH28-66	OP326456	Right-handed parallel beta-helix repeat-containing protein [*Anaerolineae* bacterium]	63.2%	MCD6284988.1
PW-pGH28-67	OP326457	Hypothetical protein [*Thermoleophilia* bacterium]	29.6%	MBN1630763.1
PW-pGH28-70	OP326460	Glycoside hydrolase family 127 protein [*Kiritimatiellae* bacterium]	79.5%	MBO7689065.1
PW-pGH28-71	OP326461	Hypothetical protein [*Kiritimatiellae* bacterium]	65.1%	MBR4171973.1
PW-pGH28-68	OP326458	Right-handed parallel beta-helix repeat-containing protein [*Phycisphaerae* bacterium]	92.1%	NLZ05743.1
PW-pGH28-69	OP326459	None		
PW-pGH28-57	OP326447	Hypothetical protein BRC67_06260 [*Halobacteriales archaeon* QH_3_68_24]	36.7%	PSP51969.1

PW-pPL1-29	OP326496	Hypothetical protein [*Sedimentisphaerales* bacterium]	71.4%	MBN1127327.1
PW-pPL1-28	OP326495	Right-handed parallel beta-helix repeat-containing protein [*Dehalococcoidales* bacterium]	63.3%	MBN2240066.1
PW-pPL1-27	OP326494	Right-handed parallel beta-helix repeat-containing protein [*Candidatus Bathyarchaeota archaeon*]	37.9%	MCJ7561073.1
PW-pPL1-16	OP326483	Right-handed parallel beta-helix repeat-containing protein [*Dehalococcoidales* bacterium]	49.0%	MBN2240066.1
PW-pPL1-13	OP326480	Hypothetical protein [*Candidatus Pacearchaeota archaeon*]	31.4%	MCK5605341.1
PW-pGH28-3	OP326393	Hypothetical protein [*Bacteroidales* bacterium]	99.9%	MCB0798852.1
PW-pGH28-10	OP326400	Glycoside hydrolase family 28 protein [bacterium]	79.1%	MBV5342714.1
PW-pGH28-12	OP326402	Glycoside hydrolase family 28 protein [*Paludibacter* sp.]	72.1%	MBP6662256.1
PW-pGH28-15	OP326405	Glycosylhydrolase family 43 [*Sedimentisphaerales* bacterium]	75.7%	MBN2513852.1
PW-pGH28-16	OP326406	Glycoside hydrolase family 28 protein [*Bacteroidia* bacterium]	73.1%	NCB68308.1
PW-pGH28-17	OP326407	Right-handed parallel beta-helix repeat-containing protein [*Bacteroidales* bacterium]	92.1%	MBN1107856.1
PW-pGH28-18	OP326408	Glycoside hydrolase family 28 protein [*Phycisphaerae* bacterium]	95.4%	MBP7053613.1
PW-pGH28-19	OP326409	Gypothetical conserved protein [*Candidatus Vecturithrix granuli*]	89.8%	GAK55202.1
PW-pGH28-20	OP326410	Hypothetical protein [*Pirellulaceae* bacterium]	76.9%	MCL4203217.1
PW-pGH28-21	OP326411	Hypothetical protein COS65_21250 [*Armatimonadetes* bacterium CG06_land_8_20_14_3_00_66_21]	62.8%	PIU91673.1
PW-pGH28-22	OP326412	Glycoside hydrolase family 28 protein [*Firmicutes bacterium*]	61.2%	MBQ7915844.1
PW-pGH28-23	OP326413	Glycoside hydrolase [*Bacteroidia* bacterium]	85.2%	NCB07128.1
PW-pGH28-24	OP326414	Hypothetical protein [*Prolixibacteraceae* bacterium]	78.6%	MBW8325277.1
PW-pGH28-25	OP326415	TPA: glycoside hydrolase [*Bacteroidales* bacterium]	84.0%	HBC79975.1
PW-pGH28-27	OP326417	Glycoside hydrolase [*Bacteroidales* bacterium]	100.0%	MCB0800470.1
PW-pGH28-29	OP326419	Right-handed parallel beta-helix repeat-containing protein [*Gloeobacteraceae cyanobacterium* ES-bin-144]	51.6%	MBC8127433.1
PW-pGH28-28	OP326418	Glycoside hydrolase [*Bacteroidales bacterium*]	90.4%	MBN1107803.1
PW-pGH28-32	OP326422	Hypothetical protein [*Phycisphaerae bacterium*]	92.1%	NLZ06394.1
PW-pGH28-35	OP326425	Glycoside hydrolase family 28 protein [*Phycisphaerae bacterium*]	95.2%	NLZ07851.1
PW-pGH28-36	OP326426	Glycoside hydrolase family 28 protein [*Prevotella* sp.]	89.6%	MBM6991671.1
PW-pGH28-34	OP326424	Glycosyl hydrolase family 28 protein [*Bacteroidales bacterium]*	79.4%	MCJ7448203.1
PW-pGH28-38	OP326428	Glycosyl hydrolases family 28 [*Verrucomicrobia bacterium* ADurb.Bin070]	98.3%	OQC31124.1
PW-pGH28-39	OP326429	Hypothetical protein [*Phycisphaerae bacterium*]	86.1%	MBP7053963.1
PW-pGH28-41	OP326431	Glycoside hydrolase family 28 protein [*Paludibacteraceae bacterium*]	77.9%	MBN2765589.1
PW-pGH28-42	OP326432	Glycoside hydrolase family 28 protein [*Bacteroidales* bacterium]	76.6%	MBK7133592.1
PW-pGH28-44	OP326434	Glycoside hydrolase family 28 protein [*Bacteroidales* bacterium]	78.7%	MBK7628679.1
PW-pGH28-45	OP326435	Glycoside hydrolase family 28 protein	100.0%	MCB9027645

		[*Bacteroidales* bacterium]		.1
PW-pGH28-47	OP326437	Hypothetical protein [*Sedimentisphaerales* bacterium]	76.5%	MBN2513767 .1
PW-pGH28-46	OP326436	Glycoside hydrolase family 28 protein [*Bacteroidetes* bacterium]	98.3%	TNF39038.1
PW-pGH28-48	OP326438	Glycoside hydrolase family 28 protein [*Bacteroidales* bacterium]	70.0%	MCE5330911. 1
PW-pGH28-49	OP326439	Glycoside hydrolase family 28 protein [*Bacteroidales* bacterium]	69.9%	MCE5330911. 1
PW-pGH28-50	OP326440	glycoside hydrolase family 28 protein [*Bacteroidales* bacterium]	73.7%	MCE5330911. 1
PW-pPL1-25	OP326492	A sorting domain-containing protein [*Bacteroidales* bacterium]	55.8%	MCE5331461. 1
PW-pPL1-24	OP326491	Hypothetical protein [*Methylobacter* sp.]	42.7%	MCL7420202. 1
PW-pPL1-22	OP326489	Hypothetical protein AMQ74_01494 [*Candidatus Methanofastidiosum methylthiophilus*]	32.2%	KYC49072.1
PW-pPL1-18	OP326485	T9SS type A sorting domain-containing protein [*Bacteroidales* bacterium]	98.0%	MBP7051750. 1
PW-pPL1-15	OP326482	Hypothetical protein [*Parabacteroides sp.* FAFU027]	66.0%	WP_2433491 03.1
PW-pPL1-14	OP326481	Hypothetical protein [*Bacteroidetes* bacterium]	66.7%	MBP1677087. 1
PW-pPL1-12	OP326479	DUF1593 domain-containing protein *[Phycisphaerae* bacterium*]*	88.7%	NLZ04906.1
PW-pPL1-5	OP326472	Hypothetical protein BGP01_06875 [*Paludibacter* sp. 47-17]	57.5%	OJX91998.1
PW-pPL1-1	OP326468	T9SS type A sorting domain-containing protein [*Catalinimonas alkaloidigena*]	52.1%	WP_0896877 57.1
PW-pGH28-63	OP326453	Glycoside hydrolase family 28 protein [*Planctomycetaceae* bacterium]	92.8%	NLS97766.1
PW-pGH28-59	OP326449	Glycoside hydrolase family 28 protein [*Clostridium estertheticum*]	59.3%	WP_2162084 74.1
PW-pGH28-56	OP326446	Hypothetical protein [*Thermoguttaceae* bacterium]	97.6%	MCL4194106. 1
PW-pGH28-54	OP326444	Hypothetical protein [*Thermoguttaceae* bacterium]	94.8%	MCL4192768. 1
PW-pGH28-53	OP326443	Glycoside hydrolase family 28 protein [*Acidobacteria* bacterium]	99.4%	NLV31532.1
PW-pPL1-26	OP326493	Pectate lyase [*Phycisphaerae* bacterium]	94.7%	MBP7052041. 1
PW-pPL1-23	OP326490	pectate lyase [*Bacteroidales* bacterium]	99.8%	MCB9028144 .1
PW-pPL1-21	OP326488	Pectate lyase precursor [*Deltaproteobacteria* bacterium]	70.7%	MBM434055 1.1
PW-pPL1-20	OP326487	Pectate lyase precursor [*Deltaproteobacteria* bacterium]	71.8%	MBM434055 1.1
PW-pPL1-19	OP326486	Pectate lyase [*Bacteroidales* bacterium]	100.0%	NLX28863.1
PW-pPL1-17	OP326484	Polysaccharide lyase [*Phycisphaerae* bacterium]	93.5%	MBP7051750. 1
PW-pPL1-10	OP326477	Pectate lyase [*Phycisphaerae* bacterium]	91.5%	MBP7051745. 1
PW-pPL1-9	OP326476	Pectate lyase [*Bacteroidales* bacterium]	80.6%	NLD63827.1
PW-pPL1-8	OP326475	Pectate lyase precursor [*Deltaproteobacteria* bacterium]	69.6%	MBM434055 1.1
PW-pPL1-7	OP326474	Pectate lyase [*Bacteroidales* bacterium]	62.3%	MCE5332852. 1
PW-pPL1-6	OP326473	Pectate lyase [*Phycisphaerae* bacterium]	80.7%	MBN2560505 .1
PW-pPL1-3	OP326470	polysaccharide lyase [*Bacteroides* sp. 51]	82.6%	WP_1631736 17.1
PW-pPL1-2	OP326469	Pectate trisaccharide-lyase precursor [*Bacteroidetes bacterium* ADurb.BinA395]	73.6%	OPZ03270.1
PW-pPL1-4	OP326471	Polysaccharide lyase [*Paludibacter* sp. SCN 50-10]	83.9%	ODT57447.1

PW-pGH28-58	OP326448	Polygalacturonase (Pectinase) [*Bacteroidetes* bacterium]	78.6%	MBP1677329.1
PW-pGH28-55	OP326445	Exopolygalacturonase [*Paludibacteraceae* bacterium]	77.1%	MBN2766841.1
PW-pGH28-52	OP326442	Exopolygalacturonase [*Bacteroidales* bacterium]	73.8%	NLS98689.1
PW-pGH28-51	OP326441	Exopolygalacturonase [*Bacteroidales* bacterium]	75.8%	NLS98689.1

Table S2. 23 pairs of primers used to amplify the 23 pectinolytic genes derived from pulp and paper wastewater treatment microbiota.

Primer names	Primer sequences
PW-pGH28-1-F	GCCTGGTGCCGCGCGGCAGCATGCGCATTGCTGGACGGTG
PW-pGH28-1-R	GGTGCTCGAGTGCGGCCGCTCTGCGCAGATACCACCAGC
PW-pGH28-2-F	GCCTGGTGCCGCGCGGCAGCATGCAGCCACTGCAGCGTGGGATTC
PW-pGH28-2-R	GGTGCTCGAGTGCGGCCGCGGGTGCGGGTGGCGCCATG
PW-pGH28-3-F	GCCTGGTGCCGCGCGGCAGCATGAGAGTGAGCAGGTTTTTGATAATG
PW-pGH28-3-R	GGTGCTCGAGTGCGGCCGCTCTGACTCTGGTTGTGCTGACAC
PW-pGH28-4-F	GCCTGGTGCCGCGCGGCAGCATGTACTTTAACAATAAACTAATTATTATCTC
PW-pGH28-4-R	GGTGCTCGAGTGCGGCCGCTTTTTTGGAATTCTCTATCATGACACGTATATC
PW-pGH28-5-F	GCCTGGTGCCGCGCGGCAGCATGAAACACACGCTCATCGTCGGGTTG
PW-pGH28-5-R	GGTGCTCGAGTGCGGCCGCGAAGGTCCAGTAGCGGTCGGGGTGC
PW-pGH28-6-F	GCCTGGTGCCGCGCGGCAGCATGAACATGACCCGATTCCAGG
PW-pGH28-6-R	GGTGCTCGAGTGCGGCCGCGCCGCGTGGTCCTAACAG
PW-pGH28-7-F	GCCTGGTGCCGCGCGGCAGCATGACGACAAGAAAACAGGTTTTCAG
PW-pGH28-7-R	GGTGCTCGAGTGCGGCCGCCCTTGCGACATTCACTTGCC
PW-pGH28-8-F	GCCTGGTGCCGCGCGGCAGCATGAACTCCCGCACAATAATAATATTAATATTA AC
PW-pGH28-8-R	GGTGCTCGAGTGCGGCCGCTATTATTGAAATTGAATTTCTATTTACTTCCAC
PW-pGH28-9-F	GCCTGGTGCCGCGCGGCAGCATGAGATTCTTCGCTTTATTTTTAATAACAATAG
PW-pGH28-9-R	GGTGCTCGAGTGCGGCCGCGGAAACTAGCCTCCAATATGTTGG
PW-pGH28-10-F	GCCTGGTGCCGCGCGGCAGCATGGTACATGATTACCTGTTTTTCAGG
PW-pGH28-10-R	GGTGCTCGAGTGCGGCCGCTTTTTTAATGTCGATGTTTTTGGAGTTTTTTCC
PW-pGH28-11-F	GCCTGGTGCCGCGCGGCAGCATGATGGTGTATAAAAATTCACGCAGA
PW-pGH28-11-R	GGTGCTCGAGTGCGGCCGCTTGATAAGAAACACCTTTATCAGCTTTCC
PW-pGH28-12-F	GCCTGGTGCCGCGCGGCAGCATGATGGTTTTTAAATCTCAGATCAAAAATTTTG
PW-pGH28-12-R	GGTGCTCGAGTGCGGCCGCTCTCACTATCTCTCCGCTTAC
PW-pGH28-13-F	GCCTGGTGCCGCGCGGCAGCATGATGACGGGTGATATGAGTGG
PW-pGH28-13-R	GGTGCTCGAGTGCGGCCGCTCGCACCTCGATGCTGTC
PW-pGH28-14-F	GCCTGGTGCCGCGCGGCAGCATGATGAACAGAATATTGATATCAACAATAC
PW-pGH28-14-R	GGTGCTCGAGTGCGGCCGCTTTATGAATAATGTCCGGACTTCC
PW-pPL1-1-F	GCCTGGTGCCGCGCGGCAGCATGAAGAACTTCCTTTCGTTGACC

PW-pPL1-1-R	GGTGCTCGAGTGCGGCCGCATTTTTGATAAATTTAAAGAGTGAGATATTATTATC
PW-pPL1-2-F	GCCTGGTGCCGCGCGGCAGCATGAAAAAACTTAGCACATTAATTTTAATTACAC
PW-pPL1-2-R	GGTGCTCGAGTGCGGCCGCTTTTTGAATTTTCATACTCGAAATCCCTTC
PW-pPL1-3-F	GCCTGGTGCCGCGCGGCATGAGTTATTGCAAAAATATTTTAGCATTTGTG
PW-pPL1-3-R	GGTGCTCGAGTGCGGCCGCTAAAAGGCTCTTTTTCTTTGCCAG
PW-pPL1-4-F	GCCTGGTGCCGCGCGGCAGCATGCAAAAGATTTTTTTATTTCTTCTGGTG
PW-pPL1-4-R	GGTGCTCGAGTGCGGCCGCAAGCAGTTTTCCTTTTTCCGCCAATG
PW-pPL1-5-F	GCCTGGTGCCGCGCGGCAGCATGAAAAAATTTTTACCTTTCTTTTTTCTTATC
PW-pPL1-5-R	GGTGCTCGAGTGCGGCCGCCAATATCATTATTTTTGCTTGATAGCG
PW-pPL1-6-F	GCCTGGTGCCGCGCGGCAGCATGATGAAAAAAGTATCAAGTATTATAACGG
PW-pPL1-6-R	GGTGCTCGAGTGCGGCCGCTTTTTCTTCGTTTTCCTTTAGGATC
PW-pPL1-7-F	GCCTGGTGCCGCGCGGCAGCATGATGTATAAATATCTCATTGTTAAATTATTT
PW-pPL1-7-R	GGTGCTCGAGTGCGGCCGCCTGTTTTCGTAATAATTCATTCAGATA
PW-pPL1-8-F	GCCTGGTGCCGCGCGGCAGCATGATGACAATATTCGCTAAATATACATG
PW-pPL1-8-R	GGTGCTCGAGTGCGGCCGCGTACGTCACTCCAGCTGC
PW-pPL1-9-F	GCCTGGTGCCGCGCGGCAGCATGATGACAAGACTATATTTCGTCATACT
PW-pPL1-9-R	GGTGCTCGAGTGCGGCCGCTTTACAAAGGCCATTTAAATATTCCTC
